# Effects of lifestyle education program for type 2 diabetes patients in clinics: study design of a cluster randomized trial

**DOI:** 10.1186/1471-2458-10-742

**Published:** 2010-11-30

**Authors:** Misa Adachi, Kazue Yamaoka, Mariko Watanabe, Masako Nishikawa, Eisuke Hida, Itsuro Kobayashi, Toshiro Tango

**Affiliations:** 1 Doctoral Course of National Institute of Public Health, 2-3-6 Minami, Wako, Saitama 351-0197 Japan; 2 Department of Technology Assessment and Biostatistics, National Institute of Public Health, 2-3-6 Minami, Wako, Saitama 351-0197 Japan; 3 Department of Human Nutrition, Graduate School of Human Ecology, Showa Women's University, 1-7 Taishido, Setagaya, Tokyo 154-0004 Japan; 4 Kobayashi Medical Clinic, Ryokujukai Cooperation, 5-27-28 Sagamiono, Sagamihara city, Kanagawa 252-0303 Japan; 5 Center for Medical Statistics, SAN Building 401, 2-9-6 Higashi Shinbashi, Minato-ku, Tokyo 105-0021 Japan

## Abstract

**Background:**

The number of patients with type 2 diabetes is drastically increasing worldwide. It is a serious health problem in Japan as well. Lifestyle interventions can reduce progression from impaired glucose tolerance to type 2 diabetes, and glycemic control has been shown to improve postprandial plasma glucose levels. Moreover, several studies have suggested that continuous interventions (combined diet and exercise) can improve the plasma glucose level and reduce dosage of hypoglycemic agents.

Although many interventional studies of lifestyle education for persons with diabetes in hospitals have been reported, only a few have been clinic-based studies employing an evidence-based lifestyle education program. This article describes the design of a cluster randomized controlled trial of the effectiveness of lifestyle education for patients with type 2 diabetes in clinics by registered dietitians.

**Methods/Design:**

In Japan, general practitioners generally have their own medical clinics to provide medical care for outpatients in the community, including those with type 2 diabetes. With the collaboration of such general practitioners, the study patients were enrolled in the present study. Twenty general practitioners were randomly allocated to each provide patients for entry into either an intervention group (10) or a control group (10). In total, 200 participants will be included in the study. The intervention group will receive intensive education on lifestyle improvement related to type 2 diabetes by registered dietitians in clinics. Lifestyle education will be conducted several times during the study period. The control group will receive information on dietary intake and standard advice on glycemic control by registered dietitians. The primary endpoint is the change from the baseline value of HbA1c at 6 months. Data on health behavior and related issues will be gathered continuously over a 6-month period.

**Discussion:**

This is the first study to evaluate lifestyle education in clinics by a cluster randomization trial in Japan. The proposed study will provide practical information about the usefulness of the intensive lifestyle improvement education program in primary care settings. The study was started in September 2007 and entry of subjects was completed in December 2010. Data on the effect evaluation will be available in 2011.

**Trial Registration:**

UMIN000004049

## Background

The number of patients with type 2 diabetes (T2D) is rapidly increasing, making T2D a serious health problem worldwide. The number of diabetic patients in Japan is increasing as a result of changes in lifestyle and the social environment. Approximately 8.9 million people are estimated to have diabetes based on a hemoglobin A1c (HbA1c) value of 6.1% or more or being recipients of treatment for T2D. Approximately 13.2 million people are at high risk for diabetes, such as those with HbA1c values of 5.6% or more but not greater than 6.1%. Thus approximately 22.1 million people have diabetes or are at high risk for diabetes [1].

Meta-analyses of RCT have shown that lifestyle interventions reduced progression from impaired glucose tolerance to T2D and that glycemic control improved postprandial plasma glucose levels after meals [2-4]. Moreover, in recent years, results of several studies suggest that continuous interventions (combined diet and exercise) can improve the plasma glucose level [5-10] and reduce dosage of hypoglycemic agents [11].

In Japan, lifestyle education for diabetes is mainly conducted in hospitals. However, providing education in outpatient clinics should be effective in increasing participation in lifestyle education by patients with diabetes. In that setting, practical dietary education would be given by registered dietitians. Therefore, to develop evidence-based lifestyle education programs by registered dietitians is important. Furthermore, to conduct effective dietary education, an assessment of nutritional intake is important. A food frequency questionnaire (FFQ) is a feasible method for this purpose. We have developed a FFQ consisting of a list of 82 foods (FFQW82) [12] for use in epidemiological studies. An advantage of the FFQW82 is that patients become aware of their own problems related to dietary habits using this instrument. It may help them to understand the specific relationship between their dietary habits and problems with glycemic control. Recently we developed an intensive lifestyle improvement education (ILIE) program to be conducted by registered dietitians in a community-based clinical setting for patients with T2D.

The aim of this study is to determine the effect of lifestyle education using the ILIE program by registered dietitians for T2D patients in clinics. Effectiveness will be determined by improvements in HbA1c values and other outcome measures.

## Methods/Design

### Study Design

In Japan, many general practitioners (GPs) have their own medical clinics (primary health centers) in which they provide outpatient medical care in the community. Many patients with T2D receive medical treatment by GPs in these clinics. With the collaboration of GPs, study patients were enrolled in the present study.

**Figure 1:**
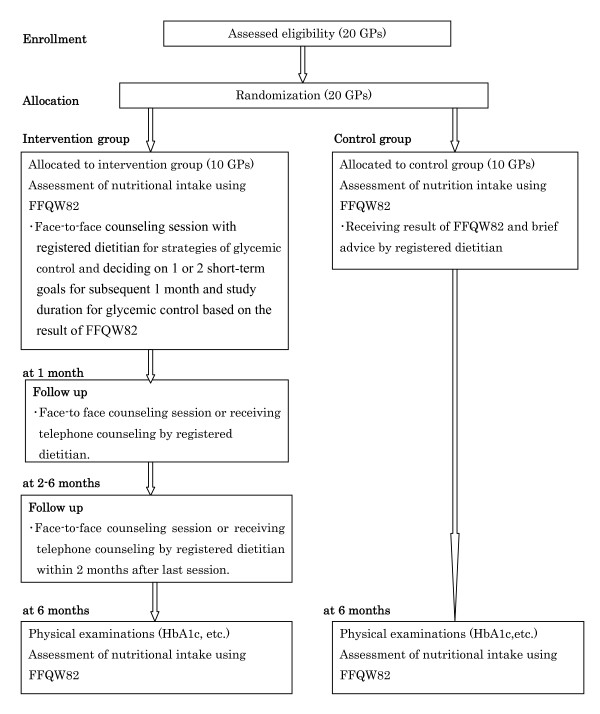
**Study design**. The file contains an overview of the study design.

The study is a cluster randomized controlled trial with two intervention arms. Each unit of the cluster is comprised of 10 patients sent by a collaborating GP. The cluster design is necessary because an individual randomized controlled study has the disadvantage in that when an individual patient is offered an intervention in the same clinic where a different intervention is offered, discussions of details of the intervention among these patients would result in an intra-correlation. The study period will be 6 months. Figure 1 is a flow diagram of the study design.

The primary endpoint is a change from the baseline value of HbA1c at 6 months. Clinical data including that on the patients' HbA1c value will be obtained from the patient's GP. Lifestyle education by face-to-face and/or telephone sessions will be conducted several times during the study period by a registered dietitian. Data on health behavior and related issues will be gathered continuously by the registered dietitian in charge over a 6-month period beginning at entry. Specifically, behavioral changes will be monitored using an assessment sheet and semi-structured interviews. The study was approved by the Medical Ethical Committee of the National Institute of Public Health in Japan in 2006 (NO.NIPH-IBRA#06005).

### Participants and recruitment

Each GP recruited 10 patients who satisfied the criteria for eligibility to participate in the study. When the participating patient gave written informed consent to his/her GP and completed the FFQW82, the informed consent document and FFQW82 were sent to the registered center by the GP and the recruitment was finished.

Study subjects are males and females from 20 to 79 years of age who have HbA1c concentrations of 6.1% or over and are receiving treatment by GPs. For subjects who take oral hypoglycemic medications or insulin, change from the baseline HbA1c level should be less than 0.5% in the previous 3 months. We excluded patients who had proliferative retinopathy over stage 3, were pregnant, had nephropathy or who had difficulty in completing written forms.

In total, a sample size of 200 subjects at high risk for or who have T2D will be recruited (see Figure 1).

### Blinding

Registered dietitians and GPs cannot be blinded to the intervention. Patients will hardly notice which intervention is assigned when he/she is informed of the education process, because only one of the two interventions is given in a specific clinic and the patient cannot be aware of what is going on at another clinic.

### Randomization

Cluster randomization was applied to avoid contamination bias [13]. Clinics are located in the metropolitan area in Kanagawa prefecture, Japan. In 2007 we recruited 24 GPs who expressed interest in participating. We provided details of the study and the standardized intervention that would be provided. We randomly assigned in turn the GP who enrolled the first high risk patient as a participant of this study to either the intervention setting or usual care setting with the use of a randomization list (random permutated blocks with block size 2) during September 2007 to December 2010. In this way, the first 20 GPs were sequentially enrolled to the study. For each GP, 10 patients were enrolled as study participants.

### Intervention and control groups

#### Intervention group

A registered dietitian in charge will make the first contact with the patient within 2 weeks from entry and hold the first session. Subjects in the intervention group will have 3 or 4 individual sessions by the registered dietitian in the 6-month period, with the second session held within a month after the first session. Also, 3 months after the first session, the participant will receive the subsequent individual session. Between the second session and the 6th month, the dietitian will contact the patient by telephone or provide a face-to-face counseling session 2-3 times to reinforce the dietary or physical activity advice and give additional support. (see Figure 1) The intensive lifestyle improvement education (ILIE) program consists of 4 strategies for glycemic control (Table 1). We developed the ILIE program based on some of the strategies described in previous studies [14-16]. Specifically, nutritional education will provide scientific information on glycemic control. Examples of advice given are to reduce energy intake at dinner and to eat one or two portions of fish/lean/meat or poultry/egg/soybean products and two portions of vegetables at each meal. A characteristic of the ILIE program is that registered dietitians can identify for patients problems of lifestyle which most often affect glycemic control by using an assessment sheet that describes matters that should be given priority for glycemic control. We made the assessment sheet by consulting evidence-based practice guidelines for treatment of diabetes in Japan [17]. Five registered dietitians decided on the priority items on the assessment sheet based on empirical evidence, with items on the upper half being more important than those on the lower half. This will help dietitians to make an objective appraisal of what practical advice to give to patients. Patients will decide on one or two short-term goals for glycemic control to be achieved in the subsequent 1 or 3 months based on the results of the FFQW82 and advice by a registered dietitian. On the other hand, for overweight or obese patients, the subject will be recommended to achieve a modest (at least 3%) weight loss. Sedentary participants will be encouraged to increase basal physical activity. We will recommend a gradual increase in physical activity in daily life rather than a formal fitness regimen or sports activity during leisure time. Several telephone follow-up calls will be made to assess progress toward the short-term goal and to offer solutions to barriers against achieving that goal. At that time, subsequent short-term goals will be decided upon.

**Table 1:** Content of Lifestyle Education Program for T2D

Content of Education	Topics
Basic information on glycemic control	Target values for body weight, HbA1c, blood pressure, blood lipids
	Diabetes complications
	Mechanism of changing of plasma glucose levels
	Effect of exercise on plasma glucose
	Needed energy intake for 1 day
	Dietary composition
Actions for glycemic control	Setting goals for glycemic control
	Solving barriers to achieving goals
Daily activities for glycemic control	Frequency and quantity of effective activity
	Setting goals for daily activity
Managing stress for glycemic control	Ways of coping with stress

##### Training of health professionals

In the 3-month period before the start of the intervention, registered dietitians received relevant training sessions with regard to the intervention protocol. Several meetings about the aspects and basic principles of the intervention protocol took place (i.e. the screening procedure, self-management principles, client centeredness, motivational interviewing, interdisciplinary collaboration, assessment tools, parts of the toolbox and referrals). Before the start of the study, registered dietitians had the opportunity to gain experience with the intervention protocol under supervision by the project team.

##### Control group

A registered dietitian in charge will make the first contact with the patient within 2 weeks from entry into the study and hold a first session. Patients in the control group will receive information on dietary intake estimated using the FFQW82 and standard advice on glycemic control by registered dietitians. They will receive the usual care provided in the outpatient clinic and can use or apply for all services previously available to them.

### Study hypothesis

The hypothesis underlying the study is that participation in the intensive dietary education group would decrease the HbA1c value by 15% from baseline (primary endpoint) in the intervention group after 6 months of education whereas such a decrease would not occur in the control group. We assumed that the HbA1c value of the control group would not change. The value of 15% increase in the HbA1c value was decided through reference to a study conducted in the USA [14] and through our experience with a previous survey [18].

### Outcome measures

The primary outcome measure is a decrease from the baseline HbA1c level after 6 months of education. The difference in the primary endpoint between intervention and control groups is the effect size of the present study. Secondary outcome measures are the differences from baseline for body mass index (BMI), waist circumference, blood pressure, fasting plasma glucose lipid profile, and the difference in energy and nutrition intakes (whole day and each meal) assessed using the FFQW82. Measurements will be made at baseline and at 6 months.

### Sample size

The sample size needed for the study was determined based on information on the effect size. We needed the participation of 20 GPs, each supplying 10 patients, to detect an effect size of 15% with SD 2.2%, an intra-class correlation of 0.1, a significance level of 5% (two-sided), a power of 80%, and equal allocation to clusters.

### Statistical analysis

Outcome measures after log transformation will be examined. Mean (SE) values of outcome measures in the intervention and control groups will be compared using the Student's *t*-test. This assumes no within clinic variance; therefore, we will add the following analysis. Namely, in order to assess within-clinic (S^2^_w_) and between- clinic (S^2^_b_) variances, linear mixed models will be used, which included a crude model (Model 1), a baseline-adjusted model (Model 2), and multivariate-adjusted model (Model 3) using individual data.

An "intent-to-treat" (ITT) approach will be performed by the Last Observation Carried Forward (LOCF) method for those subjects who drop out before the end of the 6-month intervention period.

All tests will be done with a significance level 5% (two sided). All statistical analysis will be performed using SAS version 9.2 for Windows (SAS Institute, Inc., Cary, NC, USA).

## Discussion

This paper presents a detailed description of a cluster RCT with the aim of investigating the effects of lifestyle education by registered dietitians in medical clinics for patients with T2D compared with the usual care and education provided in such clinics. One of the strengths of this study is that it is the first cluster randomization study in Japan to evaluate an intervention involving a lifestyle education program for such patients in clinics.

Twenty GPs were randomly allocated to an interventional or control group to take part in the current study between September 2007 and December 2010, with each physician recruiting 10 patients to the study. A total of 100 patients will be in each group. That these physicians applied to participate in the study indicated that they were very interested in innovations in the care of patients with T2D and at high risk for T2D. At present in Japan, T2D is managed in the general practice setting. Thus, lifestyle education on diabetes in outpatient clinics has been increasing in Japan. In this setting, GPs and nurses give brief advice on medication management and monitoring of glycemic control, but only a few dietitians are available to offer a diabetes care plan to patients with T2D. Because the success in the treatment of T2D is highly dependent on dietary habits, dietitians can be very helpful to T2D patients in mastering skills for glycemic control. Access to dietitians is easier in a clinical setting than a hospital setting.

There have been studies on lifestyle interventions in T2D in recent years including those with individual randomization [19-22] and cluster randomization designs [23-25]. The point that clinic-based studies have the possibility for contamination bias between intervention and control participants in the same clinic must be considered [13]. Thus, we felt that for our study a cluster randomization design would be best. Furthermore, in order to establish evidence-based nutrition education, examining the effects of the ILIE program presented by registered dietitians in a medical clinic-based study is important.

There are some limitations in this study design. Firstly, the success of this program is to some degree dependent on the dietitian's skill. To address this issue, we have developed a training process for the registered dietitians before the start of the randomization study. Educations in implementing the program are therefore important. Furthermore, the assessment sheet with items ranked according to priority helps standardization of advice by registered dietitians. Secondly, only patients were blinded to the groups. In order to avoid selection bias, we asked GPs to recruit all the patients in turn. Furthermore, we used HbA1c as the primary outcome, which is an objective measure. Although we cannot deny the possibility of the bias, it may be small.

The study began in September 2007 and the enrollment of patients will be completed in December 2010. The 6-month followed up period for all patients will end in June 2011. If this study appears to have positive effects, the behavioral intervention of T2D might also be implemented by other GPs in Japan.

### Progress of study

The baseline measurements started in September 2007. Data on the effect evaluation will be available in 2011.

## Abbreviations

GP: general practitioner; FFQ: food frequency questionnaire; FFQW82: food frequency questionnaire with 82 food lists; SD: standard deviation; SAS: Statistical Analysis System; BMI: Body mass index; HbA1c: hemoglobin A1c; ITT: intent-to-treat; T2D: Type 2 diabetes; LOCF: Last Observation Carried Forward

## Competing interests

The authors declare that they have no competing interests.

## Authors' contributions

MA, KY, and TT were responsible for the research questions. All authors contributed to drafting of the study protocol. MA and KY made the first draft of this paper. The other authors commented on it and approved the final version.

## Pre-publication history

The pre-publication history for this paper can be accessed here:

http://www.biomedcentral.com/1471-2458/10/742/prepub
